# Culture and Professional Success

**Published:** 1892-10

**Authors:** 


### Editorial, Etc.

CULTURE AND PROFESSIONAL SUCCESS.—The September number of *The New Review* publishes a paper on "The Relations of General Culture to Professional Success," from the pen of the late Sir Morrell Mackenzie. It has been commented upon by the *Lancet*. The article raises an issue which is pertinent to the dental profession, inasmuch as it has been proposed in the National Association of Dental Faculties that "after the session of 1892-93 four years in the study of dentistry be required before graduation." All educational methods are being subjected to stringent scrutiny. Medical schools and dental colleges have been and still are engaged in revising their curricula and extending the periods of study. Hence an article like that cited raising the great question how far general or "liberal" culture, as contrasted with special, technical and professional education is conducive to professional efficiency and success is worthy of careful attention.

The article is strongly in favor of a wide, general culture as the best preparation for the more special training which every profession demands from those who aspire to enter it. The author's grounds are various, but they may be summed up under two heads: first, that such culture confers breadth and flexibility of mind and renders the acquisition of special knowledge easy; and, secondly, that it develops a knowledge of human nature, and hence assists in enabling us to treat our clients or patients not as mere "cases" but as complex, sensitive, and highly organized men and women.

There is a strong, utilitarian wave of opinion now prevalent, and it is becoming generally assumed as an axiom that all training for a profession should be directed to the special needs and work of that profession, and that liberal study, however pleasant or refining, must, in the stern struggle for existence, make way for subjects more practical and more immediately and obviously useful.

Those most imbued with the spirit of the new era should be reminded that the preparation of the intellect for the reception of knowledge is an object hardly less great and urgent than the character of the knowledge which is imparted. Some such preparation is indispensable. The medical or dental student needs grasp and strength of mind; the capacity to note resemblances and differences; the power of determining what amount of evidence is sufficient to warrant a given conclusion; some capacity of reading character, so as to estimate correctly a patient's statements and to eliminate the personal equation. He needs to be cautioned against haste, inaccuracy, prejudice and preconceived opinions, fallacious reasoning, confused or contradictory ideas, want of tact, etc. These are formidable requirements, and the question is whether they can be met by any process less tedious and troublesome than that involved in a good preliminary education. The great danger of dental education in the present day is that raw youths with wholly unformed minds and destitute of any correct ideas regarding observation, evidence and reasoning, but with a natural aptitude for handling tools will produce specimens of workmanship which are accepted as evidences of peculiar fitness for dentistry, and they then come to imagine that little else is necessary. "The training thus commencing and ending in mechanism is discreditable, not because of its mechanism, but because being one-sided and partial it necessarily fails to accomplish that which it promises. Such training may make dental laborers, tradesmen, or artisans; but never dental artists, or scientific mechanicians; nor can the dentistry which they practice be, in any respect, identified as a branch of the art of medicine. It requires the broadest education of boyhood to counteract the necessarily narrowing influence of the professional studies of manhood; and it demands the largest possible infusion of purely scientific teaching, during professional pupilage, to correct the matter-of-fact influence of practice. In this lies the great error of American practical systems of education. They teach boyhood to take a utilitarian view of every lesson learned, and encourage young men to neglect studies in which they cannot see some prospective pecuniary value. Limitation, first in the amount of mental culture; secondly in its direction is thus made to combine with the inevitable influence of all exclusive pursuits, whether of science or business; the result is a rapid increase, in all professions, of men whose vision is limited by the narrow horizon of their special occupation, and who possess none of that large minded liberality, which is the outgrowth of a generous education. It is by such early restriction of thought and action within the narrow grooves of life's future pursuits that a merchant so often loses all power to enjoy the fruit of his toil, a physician is unknown beyond the sick-room, a surgeon contributes nothing to the cause of science, and a dentist holds no social position." R. G.

---

